# Intersecting Identities and Career-Related Factors Among College Students with Disabilities Across Ethnic Groups

**DOI:** 10.3390/healthcare13172119

**Published:** 2025-08-26

**Authors:** Si-Yi Chao, Keith B. Wilson

**Affiliations:** Early Childhood, Special Education & Counselor Education Department, University of Kentucky, Lexington, KY 40506, USA; keithbwilson@uky.edu

**Keywords:** disability identity, ethnicity identity, intersectionality, college students with disabilities, perceived career barriers, social support

## Abstract

This study explores how intersecting disabilities and ethnic identities influence key career-related factors, including career decision self-efficacy, career outcome expectations, perceived career barriers, and social support, among college students with disabilities from diverse racial and ethnic backgrounds. **Background/Objectives:** Applying social cognitive career theory (SCCT) and intersectionality frameworks, this research addresses a critical gap in understanding the unique challenges and strengths experienced by underrepresented students with disabilities in postsecondary education. **Method:** Quantitative data were collected from approximately 306 participants representing various ethnic groups, including African American, Asian American, Hispanic, and other ethnic backgrounds, alongside European American peers. **Results:** Findings revealed that underrepresented students with disabilities reported significantly stronger ethnic identity affirmation but also perceived greater career-related barriers compared to their European American counterparts. These results demonstrate the need for culturally responsive career development practices and inclusive campus environments that affirm students’ multiple identities. **Conclusions:** Implications are discussed for higher education professionals, rehabilitation counselors, disability service providers, and career counselors seeking to promote equitable career outcomes and identity-conscious support systems.

## 1. Introduction

In recent years, social justice debates have increasingly focused on making higher education institutions more accessible to historically marginalized groups of students, such as students of color, LGBTQ+ students, women in science, technology, engineering, and math (STEM) fields, students from low-income families, and most critically, students with disabilities. Colleges and universities today enroll more students with disabilities than ever before [1]. The growth in college students with disabilities from different backgrounds is a broader trend toward greater diversity on campuses throughout the nation.

National data reports a growing trend that more students with disabilities are enrolled in college and represent a wide range of racial and ethnic backgrounds. The percentage of undergraduates reporting a disability has increased significantly, rising from 10.8% in the 2007–2008 academic year to 20.5% in the 2019–2020 academic year [2,3]. This increase is due to better awareness and access to disability services at colleges. Furthermore, the characteristics of students with disabilities have changed, becoming more racially and ethnically diverse over the past two decades. The percentage of students who identified as two or more races and had a disability was highest in 2019–2020, at 25.4%. They were followed by American Indian/Alaska Native students at 23.7%, Pacific Islander students at 22.1%, Hispanic students at 21.3%, White students at 21.1%, Black students at 18.0%, and Asian students at 13.9%. These numbers reflect a clear shift from the year 2016, when the population of students with disabilities in higher education was both smaller and less diverse [2]. The proportion of Hispanic and Black students with disabilities has increased with their college attendance. At large, these trends highlight the significance of inclusive and culturally responsive services that address the unique needs of college students of color with disabilities at the postsecondary level.

The campus environment, which is increasingly multicultural but still subject to predominant cultural pressures, typically reveals larger societal dynamics through the lens of a disability and ethnic minority college student. For underrepresented students, coping with predominantly European American institutional demands is stressful and frustrating. Decision-making, interests, goals, and engagement in career tasks are all influenced by intersection of disability and racial/ethnic identity [4]. College students of color with disabilities face unique and multifaceted career development challenges rooted in systemic racism, ableism, and a lack of culturally responsive support services. The students often experience issues such as a shortage of diverse role models, career counseling that is insufficiently inclusive, and limited participation in internships and employment-based learning experiences. The structural barriers impede a minority college student with a disability in following through and retaining high career aspirations through significantly damaging self-confidence, lowering motivation, and negatively impacting career outcome expectations [5,6]. Identity development can be challenging for students with multiple marginalized identities as they navigate higher education in the complex sociocultural environment, which is characterized by power disparities, social inequalities, and intersecting forms of privilege and oppression. Multiple marginalized students might struggle to build and evolve complex selves despite constant processes of surveillance and negotiation [7]. Intersectional racial/ethnic and disability identity affirmation and the pursuit of academic and professional fulfillment require ongoing observation, constant self-reflection, and assessment of one’s own self-efficacy [6]. Moreover, social support from families, peers, mentors, and the campus is crucial in fostering resilience and perseverance, particularly when students experience discrimination or inadequate representation in mainstream career services [8,9].

Despite these challenges, few empirical studies have explored the complex perspectives of college students with disabilities from racially and ethnically diverse backgrounds. Research on the different perceptions between intersecting identities and important career-related factors is desperately needed. Implications for career counselors, disability service professionals, academic advisers, and postsecondary institutions are provided. Developing culturally responsive, identity-conscious career development programs and fostering inclusive campus climates should be emphasized to effectively address the specific needs and barriers for underrepresented college students of color with disabilities.

### 1.1. Career Decision Self-Efficacy and Outcome Expectations

The social cognitive career theory (SCCT) [10] offers a comprehensive framework for understanding the career development of individuals, especially of those with intersecting marginalized identities, such as race/ethnicity, and disability. The two main concepts that SCCT focuses on are career decision self-efficacy, which is defined as one’s belief in and confidence about their abilities to successfully make career-related decisions, and career outcome expectations, which are defined as one’s beliefs about their ability to achieve successful academic and vocational outcomes, engage in competitive employment, and progress in their career trajectory [11]. Lent et al. [10] highlighted that career self-efficacy plays a foundational role in shaping an individual’s career outcome expectations. These constructs are shaped by personal characteristics, learning experiences, and sociocultural contexts.

According to the SCCT framework, Ochs and Roessler [12] discovered that adolescents with disabilities reported much lower levels of career skills self-efficacy and career outcome expectations in comparison to their peers without disabilities. These disparities have aligned with previous studies showing that career choices, decisions, goals, and outcome expectations are more likely to differ for women, people with disabilities, and racial/ethnic minorities than the majority of White, non-disabled men in the workforce [13,14,15]. For racially/ethnically diverse college students with disabilities, the development of career decision self-efficacy and positive outcome expectations is typically undermined by multiple intersecting barriers. These may include systemic racism, ableism, limited access to role models, and a lack of culturally responsive institutional support [16,17]. Research consistently shows that underrepresented students, particularly those with intersecting marginalized identities, report lower career decision self-efficacy due to experiences of discrimination and reduced access to affirming environments [18,19]. Thus, college students with disabilities need to evaluate disability conditions in academia and career development, identify self-identity status involving the environment, and finally, believe in one’s abilities, and persist through achievement. Career decision self-efficacy is an essential ability of a college student with disabilities to make decisions and develop one’s career path.

Despite the theoretical linkage of SCCT, relatively few empirical studies have simultaneously examined both constructs, career decision self-efficacy and career outcome expectations, in the context of college students of color with disabilities. Most of the existing literature focuses on either career decision self-efficacy or outcome expectations separately, thereby overlooking the role of self-efficacy beliefs and decisions on minoritized college students with disabilities’ perceptions of their future career success. Additionally, the gap restricts our understanding of how these beliefs are intertwined with the development and the affirmation of their intersectional identities.

### 1.2. Intersectionality of Disability and Ethnic Identities

In recent years, an increasing number of researchers in vocational psychology and career development have begun to report on the experiences of individuals with intersecting social identities related to race, ethnicity, gender, disability, and sexual orientation [20,21]. Mpofu and Harley [4] pointed out that identity is a key factor in career development. The way that the students understood their disability and racial/ethnic identity played a role in their understanding of strengths, career goals, and challenges, and the way that they viewed or responded to career barriers. Building on the foundational constructs of SCCT, it is essential to examine how these theoretical principles manifest in the lived experiences of individuals with intersecting marginalized identities. In particular, understanding how disability intersects with racial and ethnic identities offers critical insight into the career development processes of underrepresented college students with disabilities.

Intersectionality, first conceptualized by Crenshaw [22], refers to how two or more identities overlap and how individuals experience compounded effects of marginalization based on dominant power structures. These intersecting identities range from race, ethnicity, gender, disability, sexual orientation, and socioeconomic status, among many others. Intersectionality entails two important implications. First, intersectionality acknowledges that social identities are jointly constructed and inseparable nature [23]. A Hispanic American with a disability that impacts mental health is an example of the intersection of ethnic and disability identities. Second, intersectionality highlights the consequences of overlapping identity, including compounded discrimination or social exclusion. As noted by Alvarado and Hurtado [24], one’s sociocultural context greatly determines how one identifies. For example, a Hispanic American with a mental health issue may be marginalized based on ethnicity and disability, respectively, while situated in the United States. However, should a Hispanic American migrate to Colombia, while potential marginalization for disability may persist, the marginalization of ethnicity may be obscured. In this situation, the Hispanic American may face new issues requiring consideration of marginalization based on gender, language, or citizenship, reflecting new potential default identifications. These affordances demonstrate the person–environment interaction central to the theory of intersectionality [23,25]. That is, identity is fluid, as shaped by place, time, and culture.

While the theoretical foundation of intersectionality continues to grow, many fields within human services and disability studies continue to view client identities as separate and distinct, rather than as connected and interacting [26]. In a systematic review of 41 peer-reviewed articles on disability identity, Forber-Pratt et al. [21] noted an average of only 24% of studies integrated or recognized intersectionality in the disability research field. In addition, half of the studies did not even report participant race. Among the remaining studies, 70% of the participants were European American; only 9.4% were African American, 8.8% Latino/a, and 11.8% in another racial/ethnic group. Very few studies examined how intersectional identities impact the psychosocial development, academic success, career pathways, or community involvement of underrepresented communities. Therefore, people with disabilities from various cultural backgrounds (e.g., people of color, women, and LGBTQ+) have their own unique needs and expectations that deserve greater attention in research and practice.

Indeed, during formative years like college, when identity development is particularly salient, the intersection of disability with race and ethnicity may significantly impact self-concept, self-esteem, and self-efficacy. Foster and Kinuthia [27] explored multicultural identity development among 33 deaf college students from underrepresented racial/ethnic backgrounds, including African American, Asian American, and Hispanic students. Their study emphasized the role that self-reflection, K–12 educational experiences, and family upbringing played in the development of disability identities. Notably, participants described identity as fluid and context-dependent, often shifting based on life transitions, peer interactions, and institutional messaging. After speaking with a college disability support provider who explained the difference, one student in the study who had previously identified as hard of hearing changed their identity to deaf. Others shared experiences when they prioritized their deaf identity over their racial identity, or the reverse, depending on the context. These narratives of participants present that identity is not just a static label but a biographical process, like a story students construct and reconstruct while they navigate different environments and evolving social expectations.

Mejia-Smith and Gushue [19] found that for Latina/o college students, ethnic identity pride was associated with greater awareness of career barriers. However, students who held stronger ethnic identity also reported higher career decision self-efficacy, which in turn was negatively associated with perceived barriers. The findings suggest that identity can be a source of both challenge and resilience. Similarly, Bounds [28] reported that among African American high school students, academic self-concept and ethnic identity were positively linked to career decision self-efficacy. The role of both cultural affirmation and academic confidence are emphasized in supporting vocational development [28]. For college students with disabilities, especially those from racial and ethnic minority backgrounds, the intersecting marginalized identities significantly influence their career development, decision-making confidence, and career outcome expectations [19,27]. Foster and Kinuthia’s [27] findings challenge practitioners to recognize that intersecting identities are not additive but dynamic and deeply contextual. They serve as lenses through which individuals experience the world, seek support, and shape their sense of self. Understanding how students of color with disabilities navigate their identities can offer valuable insight into their help-seeking behaviors, community involvement, and career pathways.

Lastly, Chao et al. [29] investigated the factors comprising ethnic identity, disability identity, perceived career barriers, and social support that influence career decision self-efficacy and outcome expectations among 312 college students with disabilities. The study found that 30.1% of the variability in career decision self-efficacy was explained by social support, ethnic identity, and disability identity. Furthermore, 56.1% of the variance in career outcome expectations was explained by career decision self-efficacy, ethnic identity, and perceived career barriers. While ethnic identity significantly predicted both career decision self-efficacy and outcome expectations, disability identity contributed to the model for self-efficacy but did not show a significant relationship with outcome expectations. However, the study did not explore whether perceptions of intersectional identities differed across ethnic groups among college students with disabilities, leaving this important dimension unaddressed. This gap signals the need to further examine how perceptions of intersecting identities and career barriers vary across ethnic groups among college students with disabilities in pursuing their career goals.

### 1.3. Career Barriers

The intersection of disability and racial/ethnic identities creates complex challenges for students’ career aspirations, career decision-making self-efficacy, and outcome expectations. While these identities contribute to professional development, they simultaneously expose students to multifaceted structural and cultural barriers that extend beyond single identity experiences [29,30]. The growing diversity in higher education brings both opportunities and challenges for students’ academic and career trajectories, particularly affecting those navigating multiple marginalized identities. College students with disabilities from racially and ethnically diverse backgrounds encounter more intricate challenges in pursuing their career goals than their European American peers. These students must navigate disability-related obstacles while simultaneously managing ethnic identity development and confronting others’ perceptions about their intersectional status. The compounded effects of marginalization create additional barriers that influence their career choices, goals, and perceived barriers [16]. Furthermore, students positioned at the intersection of disability and racial/ethnic minority status face multilayered difficulties including disability-related barriers, systemic racism, cultural stigma, and underrepresentation in academic spaces [1].

The literature reveals that ableism and racism operate differently when experienced simultaneously rather than in isolation. For example, ableism manifests differently for a Black college student with a disability compared to a White student with a disability. A Black student using a wheelchair encounters physical inaccessibility and disability-related stereotypes, but the Black student additionally experiences race-based profiling on campus, adding another layer of scrutiny from peers and faculty [31,32]. Likewise, consider a Black student with Autism Spectrum Disorder (ASD) requesting extended exam time. While White peers with similar accommodations are viewed as “hardworking individuals overcoming challenges,” the Black student might face skepticism about seeking “unfair advantages” and questions about academic belonging based on intersecting racial and ableist assumptions. Conversely, racism is experienced differently by students with disabilities than by non-disabled peers from the same racial/ethnic background. For instance, while a non-disabled Black student might face assumptions about academic preparation, A Black student with ASD encounters compounded stereotypes which are attributed to both racial assumptions about the education and learning opportunities they had before college and disability-related assumptions about cognitive ability, creating intensified perceptions of academic incapability [9,33]. These overlapping biases exacerbate exclusion from academic opportunities and career networks, demonstrating that ableism and racism are not merely additive but mutually reinforcing forces [22,34].

Research has shown that college students with intersectional identities, such as disability and race/ethnicity, manage college expectations while attempting to navigate and explore spaces that do not necessarily recognize, understand, or support their complex individual identity experiences. Additionally, perceived stigma, cultural expectations, and stereotypes about discrimination in the labor market negatively impact education and career paths [19,35]. Although the previous literature has not explicitly focused on students with disabilities, the research indicates that developing an intersectional identity [36] during the college years is an important factor in shaping career self-efficacy and outcome expectations for underrepresented students with disabilities [4,21]. Transferring from a local high school to a college or university, which often functions as a more diverse environment like a small society. For racially and ethnically diverse students with disabilities, this shift often required them to re-evaluate their sense of identity and determine where they belong and how they position themselves within the new environment [24,37]. This identity renegotiation process occurs alongside academic adjustment and career exploration, creating multiple developmental demands simultaneously.

To fully acknowledge the challenges faced by these marginalized students, it is essential to examine the varying levels of perceived career-related barriers experienced by college students with disabilities from different racial and ethnic backgrounds, both within educational institutions and across broader sociocultural systems [5,30]. This research foundation supports the critical need for appropriate career counseling and career-related development in postsecondary education. Effective career services could assist underrepresented college students with disabilities to build identity-affirming confidence, develop beliefs about their competencies for career decisions and outcomes, and eliminate perceptions of career barriers related to their intersecting identities [38,39,40].

### 1.4. Social Support

Social support is essential for the career success and well-being of college students with disabilities and those who manage intersecting disability and racial/ethnic identities. Students with disabilities are more likely to face unique challenges, including physical barriers, stigma, and the need to request accommodation [41,42]. Family, peer support, affinity groups, culturally sensitive instructors, and disability services are examples of supportive networks that can offer emotional validation, a sense of community, and guidance in navigating social and academic settings [43,44]. Students feel valued and empowered when their instructors and program faculty are understanding and accommodating [42,43]. Peer support through mentoring programs or student organizations can reduce feelings of isolation, build resilience, and foster a sense of belonging [41,44]. Positive campus climates supported by institutes provide easily accessible resources and encourage inclusive practices [41,43]. Research shows that minority students with disabilities living with strong support networks and inclusive campus climate report higher self-efficacy confidence, mental well-being, and academic engagement [43,44]. A study by Dutta et al. [45] examined career interests and goal persistence in STEM (science, technology, engineering, and mathematics) among 115 college students with disabilities, of whom 82 (71%) identified as African American and 33 (29%) as Hispanic. The study investigated several SCCT-related factors, including barriers to coping efficacy, academic self-efficacy, career outcome expectations, and social support. Their findings revealed that social support had a direct effect on STEM goal persistence and an indirect effect on academic self-efficacy. In contrast, barriers to coping self-efficacy were negatively associated with academic self-efficacy and indirectly reduced students’ interest in STEM careers. Moreover, positive career outcome expectations strongly predicted both STEM interest and goal persistence [45]. Additionally, Chao et al. [29] indicated that the intersection of social identities, the presence of adequate social support, perceptions of barriers related to social identity, and higher levels of career decision self-efficacy are crucial factors in promoting positive career outcomes among college students with disabilities. These results underscore the pivotal role that social support plays, especially for students who report low academic and career self-efficacy. Enhancing academic self-efficacy may not only boost career interest and persistence but also buffer the negative effects of identity-related barriers. Strengthening a support system and creating inclusive and affirming spaces where intersectional identities and cultural backgrounds are both acknowledged are crucial to ensuring equity and success for college students of color with disabilities in higher education [41]. Hence, there is a need to understand how college students with disabilities from diverse racial/ethnic groups perceive social support in relation to their dual identities, career development factors, and barriers they face.

### 1.5. Purpose and Significance of the Study

Despite the increasing focus on equity and inclusion in higher education, there remains limited research on how college students with disabilities navigate career development through their intersecting identities, particularly in relation to disability and race/ethnicity, and other factors. Building on the work of Chao et al. [29], they found that social support was the strongest predictor of career decision self-efficacy, followed by ethnic identity and disability identity. Additionally, career decision self-efficacy, ethnic identity, and perceived career barriers were significantly associated with career outcome expectations. The present study seeks to address how perceptions of disability identity, ethnic identity, perceived career barriers, social support, career decision self-efficacy, and career outcome expectations differ among college students with disabilities from diverse racial and ethnic backgrounds. Determination of such differences is important as the population of students with disabilities in higher education continues to become more diverse.

This study foregrounds the voices and life experiences of students with intersectional marginalized identities, therefore contributing to initiatives towards creating more inclusive and supportive learning environments. The results are expected to inform culturally responsive practice in the disability support industry, career counseling, academic advising, and school policy development. The study highlights the critical need for educators, counselors, rehabilitation and human services professionals, and disability support specialists to advance multicultural competence. It is imperative that multicultural competence be developed to make sure that all students, particularly those who are experiencing the intersection of disability, race, and ethnicity, obtain the validation, resources, and support they require to thrive in their academic and professional success.

### 1.6. Research Question

To explore these concerns, this study examines the following research question: are there significant differences among racial/ethnic groups of college students with disabilities regarding disability identity, ethnic identity, perceived career barriers, social support, career decision self-efficacy, and career outcome expectations?

## 2. Method

### 2.1. Participants and Sampling

The target population for this study was college students with disabilities enrolled in postsecondary education in the United States. Eligibility criteria included (1) current or prior enrollment in a college or university, (2) being 18 years of age or older, and (3) self-identification as having a physical or mental impairment that substantially limits one or more major life activities, as defined by the Americans with Disabilities Act (ADA). A cross-sectional survey design using a non-probability convenience sampling method was employed at two four-year public universities, one located in the midwestern region and the other in the southeastern region of the United States. This sampling method was selected due to the researchers’ prior affiliation with both institutions, which provided them with access and familiarity that facilitated participant recruitment and data collection. The total number of college students with disabilities enrolled at the two universities during the 2018–2019 academic year was approximately 2900, including about 500 students at the midwestern university and about 2400 students at the southeastern university. Eligible students were invited to participate in the study via email distributed by the Directors of Disability Support Services at each institution. A total of 306 valid participants who completed the entire questionnaire were included in the final analysis.

Among the 306 participants, 225 (73.5%) identified themselves as White/European Americans, 19 (6.2%) identified themselves as Black/African Americans, 21 (6.9%) identified themselves as Hispanics, 17 (5.6%) identified themselves as Asian Americans, and 24 (7.9%) responded other ethnicities, including multiethnicities, American Indian/Alaska Native, Arab, Hebrew, Appalachian, Pakistani, and Jewish. Among 306 participants, there were 94 male participants (30.7%), 208 female participants (68%), and 4 transgender participants (1.3%). Regarding disability onset type, 67 (21.9%) reported themselves as congenital disabilities, 87 (28.4%) reported as acquired disabilities, 123 (40.2%) reported they were not sure about their disability onset type, and 29 (9.5%) reported that they preferred not to answer. Regarding age, the majority of participants (n = 264, 86.3%) ranged 18–24 years and 13 participants (4.2%) were 25–34 years. Regarding education class, 103 (33.7%) participants reported as freshman, 75 (24.5%) as sophomores, 49 (16.0%) as juniors, 40 (13.1%) as seniors, 18 (5.9%) as master’s students, and 6 (2.0%) as doctoral students. In terms of disability type, 16 participants (5.2%) reported having physical disability, 88 (28.8%) having a mental illness and psychiatric disability, 50 (16.3%) having Attention Deficit/Hyperactivity Disorder (ADD/ADHD), 9 (2.9%) having Autism Spectrum Disorder (ASD), 23 (7.5%) having learning disabilities, 35 (11.4%) having chronic disorders, 8 (2.6%) reported having low vision or blindness, 3 (1.0%) being hard of hearing or having deafness, and 4 (1.3%) having intellectual or developmental disability (Table 1).

### 2.2. Independent Variable

The independent variable in this study was the self-reported race/ethnicity of college students with disabilities. Participants were categorized into five groups:

1. White/European American;
2. Black/African American;
3. Hispanic/Latino(a);
4. Asian/Asian American;
5. Other ethnicities, which include college students with disabilities identified themselves as Jewish, Irish, Pakistan, Arabic, and multi-races/ethnicities.

### 2.3. Dependent Variables

The six dependent variables as below were examined as the perceptions and the experiences of college students with disabilities.

1. Disability Identity;
2. Ethnic Identity;
3. Perceived Career Barriers;
4. Social Support;
5. Career Decision Self-Efficacy;
6. Career Outcome Expectations.

### 2.4. Instruments

Six dependent variables were assessed using validated instruments designed for diverse populations, with prior studies reporting reliability coefficients ranging from acceptable to high internal consistency. Each instrument was selected based on its relevance to the constructions examined in this study, which comprise disability identity, ethnic identity, perceived career barriers, social support, career decision self-efficacy, and career outcome expectations. The following sections provide a brief description of each instrument, including purpose, structure, scaling, and reliability evidence.

**Personal Disability Identity Scale (PDI).** Disability identity was measured using the PDI, developed by Hahn and Belt [46]. The PDI is an eight-item instrument that measures two dimensions, including *disability affirmation* and *disability denial*. Items are rated on a 5-point Likert scale (1 = strongly disagree to 5 = strongly agree), with reverse scoring applied to denial items. Average scores range from 1 to 5, with higher scores indicating stronger disability identity. Sample items include “I feel proud to be a person with a disability” (affirmation) and “My disability sometimes makes me feel ashamed” (denial). Previous studies reported Cronbach’s α ranging from 0.43 to 0.81 for affirmation and 0.50 to 0.79 for denial. The Cronbach’s α was 0.84 in this study, reflecting good reliability.

**Multigroup Ethnic Identity Measure—Revised (MEIM-R).** The MEIM-R adapted from Phinney [47], is a twelve-item scale assessing two dimensions: (1) ethnic identity search, which evaluates a developmental and cognitive component and (2) affirmation, belonging, and commitment, which reflects an affective component [48]. Responses use a 4-point Likert scale (1 = strongly disagree to 4 = strongly agree). No items require reverse scoring. Mean scores range from 1 to 4, with higher scores indicating stronger ethnic identity. The MEIM-R consistently shows good reliability (α > 0.80) across diverse groups [49]. The Cronbach’s α was 0.93 in this study, reflecting excellent reliability.

**Perception of Barriers Scale (POB).** Originally developed by McWhirter [50], the POB was adapted in this study to assess disability-related barriers alongside racial and ethnic barriers. A total of 13 items were used: four items measuring perceived ethnic discrimination, five items for disability-related barriers (modified from original gender items), and four items for general career barrier. Items are scored on a 5-point Likert scale (1 = strongly disagree to 5 = strongly agree), with one reverse-coded item. Higher mean scores indicate greater perceived barriers. Previous studies reported a Cronbach’s α of 0.87 for the POB [50]. The Cronbach’s α was 0.91 in this study, confirming excellent internal consistency.

**College Students with Disabilities Campus Climate Survey (CSDCC).** The CSDCC, developed by Lombardi et al. [51], assesses perceptions of social support and campus climate. In this research, 22 items were taken from five subscales, including peer support (4 items), family support (4 items), disability services (4 items), campus climate (5 items, with an additional item on ethnicity), and faculty support (5 items, taken from teaching practices and accommodation categories). Items are rated on a 6-point Likert scale (1 = never true to 6 = always true). Higher mean scores reflect higher perceived social support. Prior studies report subscale reliabilities ranging from α = 0.62 to 0.88 [51]. Convergent validity was demonstrated through correlations with GPA, self-efficacy, and campus belonging. Cronbach’s α in this study was 0.85, confirming good internal consistency.

**Career Decision Self-Efficacy Scale—Short Form (CDSE-SF).** The CDSE-SF is a 25-item scale that measures a person’s self-perceived confidence in career decision-making [52]. The CDSE-SF consists of five subscales: self-appraisal, occupational information, goal selection, planning, and problem-solving. Each subscale has five items. The participants rate their responses on a 5-point Likert scale, from 1 (no confidence at all) to 5 (complete confidence). High mean scores reflect a greater level of career decision self-efficacy. The CDSE-SF is widely validated and has demonstrated excellent internal consistency (α = 0.94) [53]. The Cronbach’s α was 0.96 in this study, reflecting excellent reliability.

**Vocational Outcome Expectations (VOE) Scale.** The VOE developed by McWhirter et al. [54], is a 12-item scale assessing expectations about future career success. This study used 10 items, omitting two that were unrelated to career goals. Responses are rated on a 4-point Likert scale (1 = strongly disagree to 4 = strongly agree), and the mean score of all items is used for scoring. Higher mean scores reflect more positive career outcome expectations. McWhirter et al. [54] reported Cronbach’s α = 0.83, and concurrent validity was supported through correlations with CDSE [36]. The Cronbach’s α was 0.94 in this study, reflecting excellent internal consistency.

### 2.5. Research Procedure

This study was approved by the Institutional Review Board (IRB) at two four-year public universities to ensure that participants’ rights and procedures complied with ethical research standards. Prior to recruitment, formal permission was obtained from the Directors of Disability Support Services at both universities. Data was collected using an online survey administered via Qualtrics. The survey was restricted to students with self-identified disabilities to ensure the appropriateness of responses. An email invitation outlined the purpose of the study, the survey link, the approximate time required to complete the survey (about 20 min), anonymous participation, assurances of confidentiality, and academic use of data. The initial email invitation and the survey link were distributed by the Directors to all students with disabilities registered with the Offices of Disability Support Services, inviting their voluntary participation. To encourage participation, the researchers requested that the Directors send two follow-up recruitment emails to the same population. The first follow-up was sent 14 days after the initial invitation, and the second follow-up was sent 14 days after the first follow-up. Participants were offered the opportunity to enter a drawing for a $5 gift card [55]. The data were collected from 15 September 2019 to 15 December 2019.

### 2.6. Data Analysis

All data were analyzed using IBM SPSS Statistics Version 28. Descriptive statistics, including frequency and percentage distributions, were used to summarize demographic characteristics such as race/ethnicity, gender, and education level. A one-way Multivariate Analysis of Variance (MANOVA) was conducted to examine whether students’ racial/ethnic backgrounds were significantly associated with differences across the six dependent variables. MANOVA was chosen due to its ability to assess multiple dependent variables simultaneously while controlling for Type I error [56,57]. Assumptions for normality, linearity, homogeneity of variance–covariance matrices, independence of observations, and multicollinearity were evaluated prior to conducting the analysis. There was no linearity, normality, multicollinearity, and homoscedasticity assumption violations in the study. Wilks’ Lambda (Λ) was used as the multivariate test statistic. Smaller Λ values indicate stronger group effects. If Box’s M test indicated a violation of the homogeneity of covariance matrices (*p* < 0.001), Pillai’s Trace was used as a more robust alternative [56]. In the event of a statistically significant MANOVA, follow-up univariate ANOVAs and post hoc tests (e.g., Scheffé test) were conducted to explore group differences.

## 3. Results

According to the analysis principles of MANOVA, the correlation values of dependent variables were 0.3 to 0.8. Because of variables somehow correlated with each other, it is appropriate to put all dependent variables in a complex for the MANOVA analysis. Before running the MANOVA, the assumptions were examined, including normal distribution, linearity, homogeneity of variances and covariances, and multicollinearity. Additionally, the assumptions of homogeneity of variance and covariance were tested for all dependent variables by using Box’s M test and the Levene’s test.

### 3.1. Box’s M Test and Levene’s F Test

The test yielded a value of Box’s *M* = 126.80, *F*(84, 13,666.94) = 1.33, *p* = 0.024. Since the *p* value of 0.024 is greater than the alpha level of 0.001, the result is not statistically significant. Therefore, the assumption of homogeneity of covariance matrices was met. The observed covariance matrices of the dependent variables in the study were equal across ethnicity groups, including European Americans, African Americans, Hispanics, Asian Americans, and other ethnicities in college students with disabilities.

In the study, the probability (*p*) value of Levene’s test of five dependent variables including ethnicity identity, perceived career barriers, social support, career self-efficacy, and career outcome expectation were larger than 0.05. Namely, these five dependent variables followed the homogeneity of error variance across different ethnicity groups. However, the disability identity variable was not homogenous across error variances in Levene’s test, *F*(4, 301) = 2.90, *p* = 0.012. Although the assumption of homogeneity of variance for the disability identity variable was violated, further examination showed that the largest standard deviation was not four times greater than the smallest, meeting the robustness guideline suggested by Mertler and Reinhart [56]. Therefore, the univariate analysis was considered robust and appropriate for use. Consequently, the MANOVA was conducted using Wilks’ Λ as multivariate test statistic to evaluate overall group differences.

### 3.2. Multivariate Analysis

A one-way MANOVA was conducted to examine whether there were significant mean differences across ethnicity groups of college students with disabilities on six dependent variables, consisting of disability identity, ethnic identity, perceived career barriers, social support, career decision self-efficacy, and career outcome expectations. The analysis revealed a statistically significant multivariate effect of ethnicity, Wilk’s Λ = 0.75, *F*(24, 1033.83) = 3.73, *p* < 0.001, partial η^2^ = 0.070 (see Table 2). Partial eta squared (η^2^), similarly to *R*^2^ in regression, indicates the proportion of variance explained by the independent variable. According to Tabachnick and Fidell [57], a value of 0.02 represents a small effect size, 0.13 a medium effect, and 0.26 a large effect. Therefore, ethnicity accounted for 7% of the variance across the six dependent variables, indicating a small to moderate effect size.

### 3.3. Univariate Analysis

A series of one-way analysis of variance on six dependent variables were conducted following the multivariate analysis to examine which specific variable had statistically significant difference across ethnicity groups. The results showed that there was a significant difference in European Americans, African Americans, Hispanics, Asian Americans, and other ethnicities in college students with disabilities on ethnic identity, *F*(4, 301) = 7.88, *p* < 0.001, partial η^2^= 0.095 (Table 3). Hence, 9.5% of the variability of the difference in ethnicity identity can be explained by college students with disabilities with different ethnicities, which had a small to medium effect size.

Due to the unequal sample size of five ethnicity categories investigated in the study, Scheffé test post hoc pairwise comparison is adequate for the unequal sample size of each ethnicity group in the study [57]. The results indicated statistically significant differences in the average scores of ethnic identity among college students with disabilities. Specifically, European American students (M = 2.66, SD = 0.57) reported significantly lower ethnic identity compared to Hispanic students (M = 3.18, SD = 0.62), mean difference = –0.52, SE = 0.14, *p* = 0.006; Black/African American students (M = 3.12, SD = 0.62), mean difference = –0.46, SE = 0.14, *p* = 0.035; and students from other ethnicities (M = 3.09, SD = 0.56), mean difference = –0.43, SE = 0.13, *p* = 0.026 (see Table 4). No significant difference was found between European American and Asian American students (M = 2.83, SD= 0.82), mean difference = −0.17, SE = 0.15, *p* = 0.85. These findings suggest that, on average, Black/African American, Hispanic, and students from other ethnic backgrounds reported higher levels of ethnic identity than their European American counterparts.

In addition, a statistically significant difference in perceived career barriers was found across ethnic groups of college students with disabilities: European American, African American, Hispanic, Asian American, and other ethnicities, *F*(4, 301) = 25.31, *p* < 0.001, partial η^2^ = 0.13. This indicates that approximately 13% of the variance in perceived career barriers can be explained by students’ ethnicity, representing a medium effect size (Table 3). Scheffé post hoc analyses revealed statistically significant differences between European American students (*M* = 2.66, *SD* = 0.57) and the following groups: Asian American students (*M* = 3.41, *SD* = 0.65), mean difference = –0.90, *SE* = 0.19, *p* < 0.001; other students (*M* = 3.16, *SD* = 0.82), mean difference = –0.65, *SE* = 0.16, *p* = 0.003; and African American students (*M* = 3.12, *SD* = 0.62), mean difference = –0.60, *SE* = 0.18, *p* = 0.025 (see Table 4). No significant difference was found between European American and Hispanic students (*M* = 2.89, *SD* = 0.95), mean difference = −0.37, *SE* = 0.17, *p* = 0.31 (see Table 5). These findings suggested that African American, Asian American, and other ethnicities college students with disabilities perceived significantly greater career barriers compared to their European American counterparts. By contrast, no significant differences were found across ethnic groups for disability identity, *F*(4, 301) = 1.2, *p* = 0.33, partial η^2^ = 0.02; social support, *F*(4, 301) = 1.4, *p* = 0.22, partial η^2^ = 0.02; career decision self-efficacy, *F*(4, 301) = 0.48, *p* = 0.97, partial η^2^ = 0.013; and career outcome expectation, *F*(4, 301) = 1.55, *p =* 0.19, partial η^2^ = 0.02 (Table 3).

## 4. Discussion

The purpose of this research was to investigate whether there are significant differences among diverse ethnic groups of students with disabilities in higher education across six main constructs: disability identity, ethnic identity, perceived career barriers, social support, career decision self-efficacy, and career outcome expectations. By integrating both the social cognitive career theory [10] and intersectionality theory [22], the results provide insight into how the intersection of disability and ethnicity influences the identity development and career-related experiences of college students with disabilities within the postsecondary education context.

### 4.1. Discrepancies of Ethnic Identity Among Groups

The first primary finding indicated a significant difference in ethnic identity among students from varying racial and ethnic groups. Specifically, Hispanic, Black/African American, and other marginalized ethnic groups had a higher degree of affirmation, engagement, and commitment of their ethnic identities in comparison with their European American counterparts. As Islam [58] emphasizes, ethnic identity is not simply determined by skin color or understood on a binary basis. Instead, it is contingent upon an individual’s cultural heritage, family descent, religious background, and sociocultural values. Disabled and non-disabled adolescents in Islam’s study [58] described their ethnic identity as being firmly rooted in intergenerational and cultural traditions.

This finding supports prior research which has demonstrated that for individuals from underrepresented groups, ethnic identity becomes more salient than disability identity when engaged in environments that are predominantly marked by standards, in this case, European American, cisgender, and non-disabled institutions [59]. The salience and dynamic development of ethnic identity among underrepresented college students with disabilities help them navigate dominant cultural contexts in higher education. For European American students, ethnic identity tends to be implicit or concealed since it is part of hegemonic cultural norms. In contrast, college students from marginalized backgrounds must engage in more conscious negotiation and affirmation of both disability and ethnic identities in contexts where such identities are either underrepresented or misunderstood within postsecondary education institutes.

From an intersectionality perspective, the identity development process becomes more complex for college students with disabilities from marginalized racial and ethnic backgrounds. These students are actively navigating identities that are frequently minimized or invisible in institutional settings rather than just confronting isolated forms of marginalization. Individuals experience social systems such as racism, ableism, sexism, and classism as interconnected and compounding systems of oppression rather than as separate forces [22]. Students who live at the intersection of marginalized racial/ethnic identities and disabilities in higher education encounter challenges in defining themselves and accessing opportunities.

This nuanced interplay between social identity and structural oppression is also illuminated by SCCT [10], which conceptualized identity-relevant experiences as part of one’s “learning experiences”. These experiences shape self-efficacy beliefs, outcome expectations, and ultimately, career decision-making. SCCT emphasizes the dynamic interaction between personal and environmental factors and sheds light on how identity development influences the formation of career decision self-efficacy and outcome expectations. For students navigating multiple marginalized identities, these learning experiences are particularly intricate, as they must simultaneously develop strategies for succeeding within systems that may not fully recognize or value their intersectional identities.

Dual negotiation of ethnic and disability identities is both a reaction to ongoing marginalization and a form of empowerment. Individuals who face persistent discrimination tend to reconstruct and redefine numerous elements of their identities to obtain access to resources, enhance self-esteem, and achieve career satisfaction [30]. Identity negotiation is crucial for determining career development, access to resources, and level of engagement in academic activities for students with disabilities from underrepresented groups. Within the SCCT framework, this negotiation is seen as a protective and adaptive behavior. Students who successfully navigate intersectional identity development in the face of discrimination may build stronger self-efficacy and develop more realistic and hopeful outcome expectations.

Collectively, the findings demonstrate that ethnic and disability identity development plays a fundamental role in framing students’ career-related beliefs and experiences. The integration of SCCT and intersectionality frameworks reveals that postsecondary institutions must move beyond one-dimensional diversity initiatives to develop comprehensive approaches that recognize and support the intersectional identity work required by students with multiple marginalized identities. Developing equitable career development outcomes requires an understanding of and a validation of such intersecting identities. Additionally, supporting the ongoing identity negotiation processes enables students to thrive in academic and professional contexts

### 4.2. Perceived Career Barriers Among Racial/Ethnic Groups

According to the second finding, students with disabilities from non-European backgrounds, including African American and Asian American students, reported greater levels of career-related obstacles in contrast to their European American peers. This pattern of differential barrier perception provides crucial insight into how intersecting marginalized identities shape career development experiences in higher education. The disparities closely align with SCCT, which emphasizes how contextual factors, such as systemic discrimination, exposure to marginalization, and limited access to social capital, contribute to career self-efficacy and outcome expectations among marginalized groups. SCCT positions that both perceived support and barriers are central in constructing individuals’ confidence and future-oriented beliefs about their career expectations [10]. For students from marginalized racial and ethnic backgrounds, systemic discrimination, limited access to social capital, and repeated exposure to marginalization create environmental conditions that heighten awareness of career barriers. This perceived barrier is not simply negative thinking; rather, it reflects a valid assessment of real structural challenges that these students encounter in academic and professional contexts.

The relationship between ethnic identity strength and barrier perception introduces an additional dimension to this finding. Research by [19] demonstrated that Latina/o students who had a strong sense of ethnic identity were not only more conscious of systemic discrimination but also more likely to identify structural career barriers. This suggests that ethnic identity development serves a dual function: while it provides cultural grounding and resilience, it also increases sensitivity to social injustice and structural inequities [4]. For students with disabilities from marginalized racial and ethnic backgrounds, this heightened awareness represents both an obstacle and a form of critical consciousness that can inform strategic career planning.

Viewed through an intersectional lens, the finding reflects how racism, ableism, and other systems of oppression intersect in complex ways that uniquely affect the career trajectories of college students with multiple marginalized identities. Supporting the framework of this study, Aquino et al. [60] found that underrepresented students with disabilities, including those identifying as Asian American, African American, Hispanic, multiracial, or LGBTQ+, were more likely to perceive discrimination than European American students with disabilities. These students frequently encountered multiple forms of marginalization, such as ableism, racism, sexism, and homophobia, reinforcing the intersectionality framework that social identities are not experienced in isolation [1]. For students who hold multiple minoritized identities, these barriers are not theoretical but are lived experiences that continuously surface in interactions with institutional systems and throughout career development efforts.

In the present study, Asian, African American, and other racially and ethnically underrepresented college students with disabilities reported significantly more perceived career barriers than their European American counterparts. According to the SCCT framework, non-European college students with disabilities who reported greater perceived career barriers than their European American peers probably lead to lower self-efficacy in making career decisions and lower expectations [29]. As a result, marginalized students may restrict their ability to pursue their goals and succeed in their careers. Also, SCCT draws attention to the mitigating role of contextual support, including affirming relationships and inclusive campus climates, that can help counteract these negative effects. These results provide empirical validation for faculty, disability services staff, diversity and inclusion professionals, and higher education leaders regarding the connection between identity-based stigma and career development challenges. Furthermore, this pattern suggests that traditional career development models, which often assume relatively uniform experiences across student populations, may inadequately address the complex realities facing students at intersectional margins.

Therefore, institutions must consider both the structural barriers and the supportive mechanisms that influence students’ career development, particularly for those with multiple marginalized identities. Synthesizing SCCT and intersectionality frameworks offers a comprehensive approach for moving beyond surface-level inclusion efforts toward structurally informed practices that acknowledge and respond to students’ complex lived experiences. Effective interventions must simultaneously address the systemic barriers these students face while providing the contextual support necessary for career development success. Culturally responsive, equity-driven interventions are essential to advance access, foster career readiness, and promote long-term success for racially and ethnically diverse college students with disabilities.

## 5. Implications for Practice: Professional and Institutional Responsibilities

### 5.1. Fostering Multicultural Counseling Competencies in Rehabilitation and Human Services

For students with disabilities navigating higher education, identity development is a multidimensional process shaped by disability, race, ethnicity, gender, and other sociocultural factors [37]. Nevertheless, disability is still too often viewed through a narrow lens of legal compliance, which is centered on accommodation rather than as a meaningful social and cultural identity [38]. This limited approach marginalizes students whose identities diverge from dominant norms and fails to fully recognize their lived experiences. Professionals in rehabilitation and human services, including disability support staff, rehabilitation counselors, and career counselors, must move beyond procedural frameworks and develop multicultural counseling competencies. These include cultural humility, intersectionality awareness, and the ability to validate students’ experiences with ableism, racism, and other systemic barriers [40,61]. Students whose social identities are repeatedly devalued may internalize stigma, experience emotional distress, and face barriers to academic and career engagement [1,62]. Rehabilitation professionals should respond not only as service providers but also as advocates and institutional change agents, supporting underrepresented students’ needs and affirming their complex identities to strengthen career decision self-efficacy and outcome expectations.

### 5.2. Advancing Culturally Responsive Career Development and Identity-Based Support

Culturally diverse students with disabilities frequently encounter systemic barriers to career opportunities, including underdeveloped mentorship networks, culturally insensitive programming, and limited internship representation [19,60]. When students must balance competing expectations in the academic, professional, and cultural domains, these obstacles can cause them to lose confidence in their ability to navigate career paths and impede their ability to achieve their career goals [39].

Career service providers, faculty, and disability support staff must collaborate to design and implement career development programs grounded in cultural responsiveness, systematic barrier navigations, and identity affirmation. This includes accessible and inclusive workshops, internships designed for diverse learners, and mentoring relationships that reflect the cultural and linguistic backgrounds of students. When students are given space to reflect on their intersecting identities and how these shape their aspirations, they are better positioned to develop resilience and career clarity [19]. Programs should also confront the broader institutional norms that discourage full participation. Institutions should recognize the systemic causes of career barriers rather than portraying them as personal constraints. This will give students the language and resources they need to confront and overcome injustices [35,61]. Culturally responsive and collaborative career development programs that affirm identity and address systemic barriers are essential for fostering inclusive participation, empowering students to explore their intersecting identities, and building resilience and career clarity.

### 5.3. Embedding Disability Culture and Intersectionality Within Institutional Inclusion Framework

Despite progress in diversity, equity, and inclusion (DEI) across higher education, disability remains underrepresented in DEI frameworks. Multicultural centers and diversity initiatives often exclude disability identity, and students are typically referred only to Disability Support Services, where the emphasis remains on documentation and compliance [38]. This practice sidelines the cultural, social, and intersectional dimensions of disability and contributes to feelings of isolation and marginalization.

Disability culture acknowledges disability as a natural and valuable aspect of human diversity and includes the shared history, values, language, artistic expression, and collective identity of individuals with disabilities [63]. As Hopson [64] explains, disability culture fosters a feeling of shared identity and passions that binds PWDs together and aids in developing and maintaining meanings, identities, and the consciousness that propels a political movement. This collective identity encompassing both cultural and sociopolitical dimensions is expressed through narrative and identity pride and is grounded in shared experiences of oppression and resilience [65]. In light of this, incorporating disability culture into campus life, we must go beyond accommodation. We need to acknowledge disability as an identity category that is equal to race, ethnicity, gender, and sexual orientation within DEI frameworks. This involves including disability perspectives in curricula, organizing disability cultural programming, and developing visible spaces that build community and pride.

To create genuinely inclusive environments, institutions must integrate disability culture and intersectionality into their DEI frameworks. Establishing Disability Cultural Centers as spaces that provide integrated wellness, identity development, vocational preparation, and social engagement can provide students with holistic support [62]. These centers should operate in partnership with Disability Resource Center, Offices of Diversity and Inclusion, Student Success, and Career Services to promote interdisciplinary collaboration and integration and dismantle structural impediments. This approach requires developing culturally responsive career services that address intersectional discrimination and mentorship programs connecting students with professionals sharing similar identity experiences.

Theoretically, integrating disability culture aligns with SCCT [10] by enhancing students’ self-efficacy, shaping positive outcome expectations, and supporting meaningful academic and career goals. This approach also reinforces intersectionality theory [22] by recognizing how disability intersects with other marginalized identities, influencing access to opportunities and resources and perceiving barriers. Creating environments where disability identity is affirmed increases students’ confidence in navigating career barriers, expands perceived career possibilities, and encourages persistence toward goals. Research demonstrates that when students’ intersecting ethnic and disability identity is affirmed, they report higher levels of belonging, self-efficacy, and engagement, which are significant predictors of career development outcomes [66]. These recommendations must be considered within the current sociopolitical climate, where DEI initiatives may face institutional and legislative resistance. Disability culture initiatives can be strong and practical ways to sustain inclusive practices while cultivating partnerships across identity groups to prevent marginalization.

Institutional staff, faculty, and administrators should receive training on disability identity, culture, and intersectionality, fostering environments where students with disabilities from all racial and ethnic backgrounds feel valued [38]. Such efforts can reduce microaggressions, improve campus interactions, and promote empathy across groups [1]. Cross-campus collaboration among peers, faculty, families, and campus leaders is essential for building supportive ecosystems where students’ identities are affirmed and their strengths acknowledged as fundamental to institutional diversity. These efforts must validate students navigating multiple marginalized identities, fostering climates that advance meaningful access, career readiness, and long-term professional success for all students.

## 6. Limitations of the Study

The first limitation of the study involves research design. The present study was a cross-sectional data collection. However, perceptions of identities, social support, career barriers, career decision self-efficacy, and outcome expectation among college students with disabilities are more likely dynamic and subject to change over time. The data was collected within a discreet period of time, which can affect the findings and the interpretations. As noted by Johnson and Christensen [67], cross-sectional designs often require large and representative samples for meaningful generalization.

The second limitation concerns the statistical power of subgroup analyses. While 250 participants identified themselves as European American, only 50 participants identified as non-European ethnicities, including African American, Asian American, Hispanic, multiethnic, Irish, and Jewish backgrounds. This distribution broadly reflects recent U.S. census data showing approximately 58% non-Hispanic White and 42% people of color. Hence, the imbalance in sample size aligns with population demographics and is not inherently problematic. That said, the relatively small size of the non-European groups may affect the robustness of statistical comparisons and limit the power to detect significant differences across ethnic groups. Even though conservative and appropriate statistical methods were employed (e.g., Scheffé post hoc analysis), the findings regarding ethnic group differences should be carefully interpreted, especially when making generalized conclusions. Given the increasing diversity of the population, future research should aim for larger and more representative samples to enhance statistical power and better reflect the broader population.

Third, the data were collected from two four-year public universities in the midwestern and southeastern regions of the United States. As a result, the present study findings cannot be generalized to college students with disabilities in all types of nationwide postsecondary education (e.g., community colleges, private universities, or institutes of technology). Future research should seek to replicate this study using more diverse institutional types and geographic regions to enhance external validity.

Fourth, participants’ responses might have been impacted by social desirability bias [68]. For instance, participants might understate perceived barriers on campus or exaggerate positive experiences with faculty support. Informed consent and confidentiality measures were taken to reduce social desirability bias, but participants might have been reluctant to express disagreement or reveal unfavorable opinions, which could potentially affect the validity of self-reported data. Researchers should remain cautious in interpreting results that may be influenced by such biases and consider incorporating methods to detect or control for social desirability in future studies.

## 7. Recommendations for Future Research

The results of this study suggest that more research is necessary to explore the extent to which students’ disability identity and ethnic identity inform the career development of college students with disabilities across multiple racial and ethnic groups. Future research also should include larger and more demographically diverse sample sizes of college students with disabilities from historically marginalized racial and ethnic backgrounds. Broader data sets allow for a better understanding of how the intersectionality of identities affects critical constructs like career decision self-efficacy, perceived barriers, social support, and career outcome expectations.

Furthermore, future research should also expand student identity development to other identity dimensions, such as, for instance, gender identity, sexual orientation, socioeconomic status, immigration background, and religious affiliation. Investigating how these constructions of layered identity affect the experience of disability and ethnicity could bring deeper insight. Understanding of identity nuance can emphasize how students experience and contend with structural and cultural barriers in higher education and beyond: for example, how a Black woman with a disability engages in the college, and then how she navigates the intersectionality of disability, ethnicity, and gender identity during the workforce experience.

Additionally, future studies should explore the potential effects of different university attendance modes (in-person vs. e-learning) on students’ experiences with identity development and career development. For students with disabilities from diverse ethnic backgrounds, the distinction between in-person and online learning environments may be pertinent because these settings may present unique opportunities and difficulties for identity affirmation and career efficacy and career outcomes [69,70,71,72]. In-person learning settings might provide more chances for mentoring and community development, but they might also come with extra challenges because of social isolation, campus climate and physical accessibility. On the other hand, online learning has the potential to improve accessibility and offer flexible accommodations. However, it may limit opportunities for meaningful social connections and access to culturally inclusive and affirming spaces, both of which are fundamental for the development of intersectional identities [70,71]. Promoting more inclusive and efficient support systems that address the individual needs of racially and ethnically diverse students with disabilities across various educational delivery formats is critical. This effort may be facilitated by recognizing how students’ perceptions of career barriers and success, social support, and intersectional identity affirmation are influenced by their mode of university attendance.

Lastly, research should not be limited to college experience. Extending research into the workforce may illustrate how identity and intersectionality continue to shape the scope of experience concerning access to career opportunities, perceptions of organizational inclusion in the workplace, and sustainable career satisfaction within the life course of individuals with disabilities. The results of future studies may resonate with rehabilitation professionals, career counselors, and higher education agents and serve to strengthen current efforts to redefine practices supportive of identity development, validate the lived experience, and dismantle systemic inequities.

## 8. Conclusions

This study illuminates the complex and intersectional influences of disability and ethnic identities on the career-related factors of college students with disabilities across racial/ethnic groups. Two critical patterns were identified. First, students from underrepresented groups reported stronger affirmation and engagement with their ethnic identities than their European American counterparts. Second, they are more likely to perceive career-related barriers compared to those who are European Americans. These patterns highlight the paradox faced by many marginalized students. While ethnic identity can serve as a source of strength, pride, and resilience, it is also associated with a greater awareness of systemic inequities in both educational and career environments.

The findings underscore the need for postsecondary institutions to adopt more nuanced, identity-conscious approaches to career development. One-size-fits-all services fall short in addressing the layered challenges faced by students who navigate the compounded effects of ableism and racism. Instead, career services, academic advising, and campus support systems must be culturally responsive, inclusive, and affirming of students’ intersecting identities. This transformation is critical for college students with disabilities from racially and ethnically underrepresented backgrounds. Professionals and institutions need to rethink disability not simply as a category for accommodation but as a dynamic social identity that intersects with race, gender, and culture. Integration of identity-affirming, culturally responsive, and collaborative strategies with career development and student support in institutions helps to reduce barriers and enables students to realize their full academic and professional potential.

## Figures and Tables

**Table 1:** Demographic data of participants.

	n	Valid Percent
Race/Ethnicity	306	100%
White/European	225	73.5%
Black/African	19	6.2%
Hispanic	21	6.9%
Asian	17	5.6%
Others	24	7.9%
Gender	306	100%
Female	208	68.0%
Male	94	30.7%
Transgender	4	1.3%
Disability Onset	277	100%
Congenital	67	21.9%
Acquired	87	28.4%
Not sure	123	40.2%
Age	295	100%
18–24	264	86.3%
25–34	13	4.2%
35–44	9	2.9%
45–54	3	1.0%
55 or older	6	2.0%
Class	291	100%
Freshman	103	34.8%
Sophomore	76	25.7%
Junior	49	16.6%
Senior	41	13.9%
Master	19	6.4%
PhD	8	2.7%
Disability Type	260	100%
Physical Disability	16	5.2%
Mental Health Issues/Emotional Disability/Psychiatric Disability	88	28.8%
Attention Deficit/Hyperactivity Disorder (ADD/ADHD)	50	16.3%
Autism Spectrum Disorder	9	2.9%
Learning Disability	23	7.5%
Chronic Disorders (e.g., Rare Diseases, Diabetes, Sickle Cell Disease, etc.)	35	11.4%
Low Vision/ Blindness	8	2.6%
Hard of Hearing/ Deafness	3	1.0%
Brain Injury	6	2.0%
Temporary Disability	15	4.9%
Epilepsy	3	1.0%
Intellectual/Developmental Disability	4	1.3%

**Table 2:** Multivariate tests ^a^ of variance table on ethnicity groups.

Effect		Value	*F*	Hypothesis *df*	Error *df*	*p*	Partial η^2^
Intercept	Pillai’s Trace	0.98	2065.39 ^b^	6.00	296.00	0.000	0.98
	Wilks’ Lambda	0.02	2065.39 ^b^	6.00	296.00	0.000	0.98
	Hotelling’s Trace	41.87	2065.39 ^b^	6.00	296.00	0.000	0.98
	Roy’s Largest Root	41.87	2065.39 ^b^	6.00	296.00	0.000	0.98
Ethnicity	Pillai’s Trace	0.26	3.51 ***	24.00	1196.00	0.000	0.07
	Wilks’ Lambda	0.75	3.73 ***	24.00	1033.83	0.000	0.07
	Hotelling’s Trace	0.32	3.94 ***	24.00	1178.00	0.000	0.07
	Roy’s Largest Root	0.27	13.42 ^c^***	6.00	299.00	0.000	0.21

^a^ Design: Intercept + Ethnicity. ^b^ Exact statistics. ^c^ The statistic is an upper bound on *F* that yields a lower bound on the significance level. *** *p* < 0.001.

**Table 3:** Tests of between-subjects effects on ethnicity groups.

Source	Dependent Variable	Type III *SS*	*df*	*MS*	*F*	*p*	Partial η^2^
Corrected Model	Disability Identity	2.70	4	0.68	1.15	0.332	0.015
	Ethnicity Identity	11.13	4	2.78	7.87 ***	0.000	0.095
	Perceived Career Barrier	25.31	4	6.33	11.26 ***	0.000	0.130
	Social Support	2.90	4	0.73	1.44	0.221	0.019
	Career Decision Self-Efficacy	1.94	4	0.48	0.97	0.425	0.013
	Career Outcome Expectation	1.51	4	0.38	1.54	0.189	0.020
Error	Disability Identity	176.60	301	0.59			
	Ethnicity Identity	106.37	301	0.35			
	Perceived Career Barrier	169.18	301	0.56			
	Social Support	151.73	301	0.50			
	Career Decision Self-Efficacy	150.39	301	0.50			
	Career Outcome Expectation	73.41	301	0.24			
Corrected Total	Disability Identity	3156.66	306				
	Ethnicity Identity	2459.67	306				
	Perceived Career Barrier	2391.87	306				
	Social Support	5340.25	306				
	Career Decision Self-Efficacy	4607.37	306				
	Career Outcome Expectation	3299.25	306				

*** *p* < 0.001.

**Table 4:** Scheffé post hoc comparison table in ethnic identity of ethnicity groups.

(I) Ethnicity	(J) Ethnicity	ΔMean (I-J)	*SE*	*p*	95% Confidence Interval (CI)
White/European	Black/African	−0.46 *	0.14	0.035	[−0.90, −0.02]
	Hispanic	−0.52 **	0.14	0.006	[−0.94, −0.10]
	Asian	−0.17	0.15	0.851	[−0.64, 0.29]
	Other ethnicities	−0.43 *	0.13	0.026	[−0.82, −0.03]
Black/African	White/European	0.46 *	0.14	0.035	[0.01, 0.90]
	Hispanic	−0.06	0.19	0.998	[−0.65, 0.52]
	Asian	0.29	0.20	0.724	[−0.33, 0.90]
	Other ethnicities	0.03	0.18	1.000	[−0.53, 0.60]
Hispanic	White/European	0.52 **	0.14	0.006	[0.10, 0.94]
	Black/African	0.06	0.19	0.998	[−0.52, 0.65]
	Asian	0.35	0.19	0.519	[−0.25, 0.95]
	Other ethnicities	0.10	0.18	0.990	[−0.45, 0.65]
Asian	White/European	0.17	0.15	0.851	[−0.29, 0.64]
	Black/African	−0.29	0.20	0.724	[−0.90, 0.33]
	Hispanic	−0.35	0.19	0.519	[−0.95, 0.25]
	Other ethnicities	−0.25	0.19	0.771	[−0.84, 0.33]
Other ethnicities	White/European	0.43 *	0.13	0.026	[−0.03, 0.82]
	Black/African	−0.03	0.18	1.000	[−0.60, 0.53]
	Hispanic	−0.10	0.18	0.990	[−0.65, 0.45]
	Asian	0.25	0.19	0.771	[−0.33, 0.84]

*Note.* Based on observed means. The error term is Mean Square (Error) = 0.244. * *p* < 0.05. ** *p* < 0.01.

**Table 5:** Scheffé post hoc comparison table in perceived career barriers of ethnicity groups.

(I) Ethnicity	(J) Ethnicity	ΔMean (I-J)	*SE*	*p*	95% CI
White/European	Black/African	−0.60 *	0.18	0.025	[−1.16, −0.05]
	Hispanic	−0.37	0.17	0.313	[−0.90, 0.16]
	Asian	−0.90 ***	0.19	0.000	[−1.48, −0.31]
	Other ethnicities	−0.65 **	0.16	0.003	[−1.15, −0.15]
Black/African	White/European	0.60 *	0.18	0.025	[0.05, 1.16]
	Hispanic	0.23	0.24	0.922	[−0.51, 0.96]
	Asian	−0.29	0.25	0.847	[−1.07, 0.48]
	Other ethnicities	−0.05	0.23	1.000	[−0.76, 0.67]
Hispanic	White/European	0.37	0.17	0.313	[−0.16, 0.90]
	Black/African	−0.23	0.24	0.922	[−0.96, 0.51]
	Asian	−0.52	0.24	0.339	[−1.28, 0.24]
	Other ethnicities	−0.27	0.22	0.828	[−0.97, 0.42]
Asian	White/European	0.90 ***	0.19	0.000	[0.31, 1.48]
	Black/African	0.29	0.25	0.847	[−0.48, 1.07]
	Hispanic	0.52	0.24	0.339	[−0.24, 1.28]
	Other ethnicities	0.25	0.24	0.895	[−0.49, 0.98]
Other ethnicities	White/European	0.65 **	0.16	0.003	[0.15, 1.15]
	Black/African	0.05	0.23	1.000	[−0.67, 0.76]
	Hispanic	0.27	0.22	0.828	[−0.42, 0.97]
	Asian	−0.25	0.24	0.895	[−0.98, 0.49]

*Note.* Based on observed means. The error term is Mean Square (Error) = 0.244. * *p* < 0.05. ** *p* < 0.01. *** *p* < 0.001.

## Data Availability

The original contributions presented in this study are included in the article. Further inquiries can be directed to the corresponding author.
